# Commissioning and initial experience with the first clinical gantry‐mounted proton therapy system

**DOI:** 10.1120/jacmp.v17i2.5868

**Published:** 2016-03-08

**Authors:** Tianyu Zhao, Baozhou Sun, Kevin Grantham, Leith Rankine, Bin Cai, Sreekrishna M. Goddu, Lakshmi Santanam, Nels Knutson, Tiezhi Zhang, Michael Reilly, Beth Bottani, Jeffrey Bradley, Sasa Mutic, Eric E. Klein

**Affiliations:** ^1^ Department of Radiation Oncology Washington University School of Medicine St. Louis MO USA

**Keywords:** proton, commissioning, passive scattering

## Abstract

The purpose of this study is to describe the comprehensive commissioning process and initial clinical experience of the Mevion S250 proton therapy system, a gantry‐mounted, single‐room proton therapy platform clinically implemented in the S. Lee Kling Proton Therapy Center at Barnes‐Jewish Hospital in St. Louis, MO, USA. The Mevion S250 system integrates a compact synchrocyclotron with a C‐inner gantry, an image guidance system and a 6D robotic couch into a beam delivery platform. We present our commissioning process and initial clinical experience, including i) CT calibration; ii) beam data acquisition and machine characteristics; iii) dosimetric commissioning of the treatment planning system; iv) validation through the Imaging and Radiation Oncology Core credentialing process, including irradiations on the spine, prostate, brain, and lung phantoms; v) evaluation of localization accuracy of the image guidance system; and vi) initial clinical experience. Clinically, the system operates well and has provided an excellent platform for the treatment of diseases with protons.

PACS number(s): 87.55.ne, 87.56.bd

## I. INTRODUCTION

Proton therapy has been known for the capability of delivering highly precise radiation doses to tumor volumes while protecting healthy tissue from radiation side effects for better outcomes.^1^, ^2^ However, this advanced technology has not been widely accepted because of: i) cost, exceeding $150 million for multiroom systems, ii) the space required to host accelerator, beamline, transport systems, and treatment rooms, and iii) complex delivery systems that require engineers specially trained and certified to run and maintain.

A compact proton therapy machine, specifically a single‐room proton therapy unit, is appealing to hospitals with a population of regional patients who benefit from this technology, based on current clinical evidence. The benefits of a single‐room system include reduced cost for i) the machine, ii) space, due to smaller footprint, iii) the construction and installation, and iv) maintenance, due to lower system complexity and power consumption. A compact system is much easier to integrate with the rest of the hospital geometrically and administratively, instead of being detached at remote location. It improves the flexibility of moving patients between proton rooms or from proton service to adjacent IMRT service in the event of a system breakdown. A compact system operates similar to a photon system as the beam is no longer orchestrated among multiple rooms by a team of engineers With a lower financial barrier, the demand for single‐room systems is expected to grow rapidly as more institutions can practically support such technology.

The world's first single‐room proton therapy system, the Mevion S250 (Mevion Medical Systems, Littleton, MA) was installed and commissioned in the S. Lee Kling Proton Therapy Center at Barnes‐Jewish Hospital in St. Louis, MO, USA. The system has been in clinical operation since the December of 2013. In this study, we present the comprehensive commissioning process and initial clinical experience with the Mevion S250.

## II. MATERIALS AND METHODS

The Mevion S250 system includes a synchrocyclotron (1.8 m in diameter and 22 tons in weight) mounted on a gantry that rotates from $-5^{\circ }$ to 185° around isocenter. A pair of annular superconducting coils made of Nb‐Sn superconductor and a pair of shaped ferromagnetic poles are used to generate a solenoid magnetic field that peaks at 8.7 Tesla at the center of the synchrocyclotron to produce a bundle of protons with nominal energy of 250 MeV. The coils, hosted by a stainless steel bobbin inside a cryostat, are cooled to 4K by cyrocoolers connected to liquid helium. A magnetic regenerator is placed close to the extraction point to produce a bump in the magnetic field that disrupts the vertical focusing of protons. Angle and pitch of proton orbits are altered toward the extraction channel.

The unique design in mounting the synchrocyclotron on the gantry eliminates the need for a transportation beamline and further reduces the requirement on space. However, the output and energy are impacted. As the gantry rotates, the gravity on the superconducting coils can shift the magnetic field by tenths of a millimeter with respect to the magnetic field regenerator designed to deviate protons into the extraction channel. To compensate for the gantry‐angle‐dependent energy fluctuations of protons into the extraction channel, a variable‐thickness wedge is introduced at the entrance to the extraction channel to fine‐tune the proton energy. Although this mechanism produces consistent mean energy output at all gantry angles, the energy spectrum differs slightly as protons go through various thickness of scattering material. As no energy selection system is present downstream in the beamline to filter out undesired energies, the variations in energy spectrum could have a direct impact on the monitor unit (MU) chamber and cause moderate output dependence on gantry angle. This effect has to be evaluated in commissioning, and accounted for in determination of output for each treatment field.

The beam extracted at 250 MeV is adjusted to the energy required for the treatment by absorber wheels that are made of graphite, and a pair of coarse energy absorber and a fine absorber that are made of Lexan. However, a magnetic analyzer is not present in the beamline to maintain a tight energy width after being degraded. This design is different from commercially available models from other vendors. Its dosimetric impact needs to be evaluated at the entry region and distal falloff.

Twenty‐four options are available for treating targets with range up to 32 cm with maximum field size of 14 cm, and up to 25 cm in depth with maximum field size increased to 25 cm. The beam‐shaping system includes a first scatter with 21 steps, a pair of alternate coarse range absorbers, a fine range absorber, three secondary scatters and 14 range modulation wheels. Steps in the modulation wheels are compensated for scattering with an appropriate ratio of lead and Lexan. All options are categorized into three groups, “small,” “deep,” and “large,” based on maximum field size and range. “Small” options treat targets up to 20 cm in depth with a maximum field size of 14 cm and modulation width from 2 cm to the range. “Deep” options treat targets with depth less than 32 cm but larger than 20 cm. The maximum field size of deep options is 14 cm and the maximum modulation width is limited to 10 cm. The deep options are mainly designed for prostate treatment. “Large” options treat targets up to 25 cm in depth with maximum field size of 25 cm and modulation width up to 20 cm. Options in the same group share the same secondary scatter. The nominal SAD of the machine is 200 cm.

### A. CT Calibration

Conversion from Hounsfield units (HU) to relative proton stopping power ratio (PSPR) plays a vital role in proton therapy. General perception of uncertainty of 3.5% on range is accepted and applied widely in proton therapy due to the uncertainty associated with CT imaging and conversion from HU to PSPR. It has been found that stoichiometric CT calibration is more precise than the tissue substitute calibration.^(3)^ Following Schneider's original implementation,^(4)^ an electron density phantom CIRS model 062^(5)^ (Computerized Imaging Reference Systems Inc., Norfolk, VA) was selected for evaluation of HU vs. PSPR due to its popular availability and easy access to the published data on substitutes’ composition. The phantom was made of an inner cylindrical part (head) and an outer ring (body). The base material of the phantom was water‐equivalent plastic with holes to accommodate 17 inserts. The dimensions of the phantom were 33 cm in width and 27 cm in height. All tissue substitutes were built with physical and electron densities similar to the recommendations of ICRU Report 44.

To evaluate the uncertainty in our CT calibration approximately, we did an inter‐institute comparison with the institutional calibration curves employed in three other proton institutions: University of Pennsylvania, MD Anderson Cancer Center, and ProCure Oklahoma. The same phantom with inserts in exactly the same arrangement was circulated through all participating institutions. After the phantom was scanned with available CT protocols for proton therapy in participating proton institutions, the acquired CT images and CT calibration curves were collected through a secure FTP server. However, CT calibration curves from all participating institutions could not be compared directly because the CT scanners involved in building the calibration curves were different in terms of manufacture, model, and energy. The PSPRs of the inserts were instead determined by applying the corresponding institutional CT calibration curves on the collected CT images, and plotted with HUs from our institution. This process rebuilt CT calibration curves from all participating institutions on the same CT scanner for direct comparison. Assuming the average of all four calibration curves the ground truth, range uncertainties from the variations of CT calibration were evaluated on our clinical plans. This process transferred variations in CT calibration into range variations. It is estimated that the range uncertainty is ±0.5% from imaging and calibration, and ±0.5% from CT conversion to tissue if ionization energy is excluded.^(6)^ We expect our range uncertainty from the CT calibration alone is close to that estimation. Since we were only interested in comparing PSPRs obtained with the active CT protocols used in the participating institutions, where the selection of kVp, FOV, slice thickness, and filter matched the parameters used for commissioning their CT calibration curves, the impact of the variations in institutional CT protocols was not studied and reported in this manuscript.

The CT calibration was also tracked on historic data from 2008 to 2014 using measurements from annual QA tests. The purpose of this assessment was to evaluate the long‐term reliability of CT calibration for proton therapy, which, if understood, would help to decide the frequency of quality assurance for the CT scanner. A replacement of X‐ray tube was performed in 2011, which offered the best opportunity to check the stability of the CT scanner.

### B. Beam Data

The general guideline for acquiring beam data for photon beam commissioning has been described in the report of AAPM Task Group 106.^(7)^ Many aspects apply to proton beam commissioning. Two special considerations apply to the Mevion S250.

First, the acquisition time for each measurement point has to be integrated over 2.08 s in order to allow the beam spot to be distributed equally over a rotating range modulation wheel (RMW). This period, coming from the interplay between the pulse frequency and modulation wheel rotation, allows the beam spot to be distributed evenly on the RMW. Otherwise, significant measurement uncertainties would be observed. However, this increases time for data acquisition.

Second, the output dependency on gantry angle has to be measured due to the presence of a fine wedge that compensates for the shifting of magnets during gantry rotation.

The beam model was commissioned in an Eclipse V11.0.30 (Varian Medical Systems, Palo Alto, CA), which employs a pencil‐beam algorithm.^(8)^ As all TPS commercially available use similar algorithms with slight differences in the modeling of Bragg peak and the calculation of spread‐out Bragg peak (SOBP), the results and discussion in this study apply generically.

For commissioning the pencil‐beam algorithm for passive scattering, four sets of measurements are required. They include (i) percent depth dose in water, (ii) longitudinal profile in air under a open block, (iii) lateral profiles in air under a half‐block, and (iv) lateral profiles in air without blocking.

**Figure 1:**
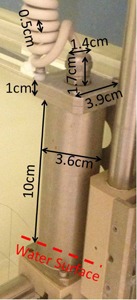
The dimensions of the moving parts that hold the chamber holder. There is a pair of the moving parts on both sides of the water tank. The dashed line indicates the water surface in our setup.

Percent depth‐dose curves were acquired using a 3D scanning tank (Blue phantom, IBA Dosimetry America, Bartlett, TN) at nominal source‐to‐surface distance (SSD) of 200 cm with water surface leveled at radiation isocenter, using a parallel‐plate chamber (PPC05, IBA Dosimetry). The PPC05 chamber has a sensitive volume of 0.046 cc with 9.9 mm in diameter and 0.6 mm in electrode spacing. An open ring aperture was used for all depth‐dose measurements to minimize collimator scatter. The entrance window of the PPC05 is made of air‐equivalent plastic C‐552 with physical thickness of 1 mm. A shift of 2.2 mm away from the source was applied on the acquired depth‐dose curve to account for i) 1.55 mm downstream shift of the effective measurement point at the inner surface of the entrance window, and ii) 0.65 mm rise of water surface after the moving parts holding the chamber holder submerged under the water surface. The dimensions of the moving parts are illustrated in Fig. 1.

The width of a pristine Bragg peak is defined at the 90% of the peak dose. Distal penumbra is defined as the distance from the 80% to the 20% in the distal falloff region. Both properties were measured and reported as they provided important information on range straggling and energy spread of protons.

Source size was determined by acquiring profiles in air at various distances from the nominal source position under half‐beam block using edge detector (Sun Nuclear Corporation, Melbourne, FL). The diode in the edge detector had a dimension of 0.8 mm in both width and length, making it ideal for measuring the sharp dose gradient. The snout position for all lateral profiles was fixed to 40 cm. The source size was modeled as a function of residual range of proton, which was achieved with various nozzle‐equivalent thicknesses (NET) by setting the modulation wheel at various steps.

Virtual source‐axis‐distance (VSAD) was determined by acquired profiles in air at transverse planes, 20 cm upstream from the machine isocenter, along isocenter, and 20 cm downstream from the machine isocenter under a square block using the edge detector.

Effective source‐axis‐distance (ESAD) was determined by acquired longitudinal profiles along the central axis using a cylindrical type ionization chamber (FC65, IBA Dosimetry). Measurements were taken at 11 points equally distributed around the machine isocenter on a span of 40 cm. The ESAD was calculated by fitting measurements according to the inverse square law. The ESAD was a function of residual range of proton, as well.

### C. Dosimetric commissioning

Validation measurements were taken for all 24 options using open fields. They included percent depth dose along the central axis and transverse profiles at various depths (middle of SOBP, one‐third of modulation width upstream, and downstream from the middle of SOBP). Measured doses were compared to predictions from the TPS. We performed 1D gamma analysis using 3%/1 mm criteria and deemed pass with results larger than 90%. The use of 1D gamma analysis was mainly used as a metric for evaluating the accuracy of beam modeling on SOBP except the distal falloff region. Any discrepancy larger than 3% on the proximal shoulder or larger than 5% in the entrance region was tuned by adjusting the partial shinning correction^9^, ^10^ and entrance region correction.

As historically noted, prediction of output and monitor unit (MU) is not supported in current treatment planning systems. Although our treatment planning system was fully commissioned to ensure accurate calculation of dose distribution, MUs are assigned to each field by explicit measurement. A verification plan was generated for each beam by duplicating the beamline onto a digital water phantom that mimicked the measurement phantom. Apertures were maintained and the range compensator was removed in the verification plans to eliminate the perturbation in dose measurement from compensator scattering. The treatment isocenter was set in the middle of SOBP where the dose output (cGy / MU) was measured. Measurements were performed with same geometry and beam parameters. A standard 10 cm×10 cm square aperture and a 0.6 cc cylindrical ionization chamber were used for all measurements, except fields with radius less than 2 cm. Special measurements for small fields were taken with the corresponding apertures in place and a small cylindrical ionization chamber due to its small sensitive volume. In addition, a MU prediction model based on the work by Kooy et al.^(11)^ was developed as a secondary check for measurements.^(12)^ The input parameters of the MU prediction model were range and modulation width. The model predicted the output by fitting all measured data. The accuracy of the prediction is expected to increase with the accumulation of additional measurements.

An inhomogeneous phantom with half‐bone‐half‐water interface was used to evaluate the TPS regarding heterogeneous media. The physical thickness of the bone slab was 2 cm and the relative stopping power ratio was 1.63. Profile was acquired on the transverse plane at the middle of SOBP in water with a pinpoint chamber (CC04, IBA Dosimetry). A proton field with range 32 cm and modulation width 10 cm was used for the test. It presented the worst‐case scenario as the measurement plane was 27 cm from the bone‐water interface, maximizing the width and amplitude of the heterogeneity in dose distribution.

The Imaging and Radiation Oncology Core (IROC) performed further validation via an on‐site audit as well as off‐site dosimetry verification by anthropomorphic phantoms irradiation. The on‐site visit reviewed all the aspects of our practice, including CT calibration, treatment planning, delivery, QA, dosimetry, and workflow. In addition, dosimetric validation was performed on anthropomorphic phantoms representing four clinical sites including craniospinal, prostate, brain, and lung.^13^, ^14^ Irradiation of the IROC phantoms served both as an admission to NCI‐funded cooperative group clinical trials and a stringent test of our institute's capability of planning and delivering treatments with heterogeneity correctly accounted for.

### D. Imaging guidance and 6D robotic couch

The Mevion S250 proton therapy unit is equipped with a 6D robotic couch and image guidance system (Verity). Setup images were provided by a pair of orthogonal planar X‐ray imagers with sources embedded in lateral wall and floor. The patient alignment process allows corrections in six degrees of freedom: translation {x,y,z}, pitch, roll, and yaw {θ,ϕ,ψ}. Geometric accuracy of couch corrections and imaging vs. radiation isocenter coincidence were quantified before clinical implementation. In addition, a gantry star shot and a couch star shot were performed to evaluate the isocentricity of gantry and couch rotation around radiation isocenter.

A commercial phantom with 16 2 mm tungsten BBs was mounted rigidly on the couch and imaged with CT. Seventeen rigid translations/rotations of known magnitude were digitally applied to the original CT image using commercial software, initially validated with Varian's OBI system. For each altered image, phantom was mounted on robotic couch in original position, then Verity 2D:2D match — posterior‐anterior (PA), and left lateral (LLAT) — was performed using DRRs from the altered images. Corrections were recorded and applied. The phantom was imaged a second time and residual corrections recorded. Physical measurements verified that applied couch corrections coincided with both physical couch shifts/rotations and known CT image translations/rotations. Additionally, imaging vs. radiation isocenter coincidence was quantified over couch treatment angles ($\pm 90^{\circ }$ from the setup position) using radiochromic film and an image‐guided couch star shot. The PA and LLAT kV radiographs were taken before each beam was delivered to verify imaging/radiation isocentricity.

## III. RESULTS

### A. CT calibration

**Figure 2:**
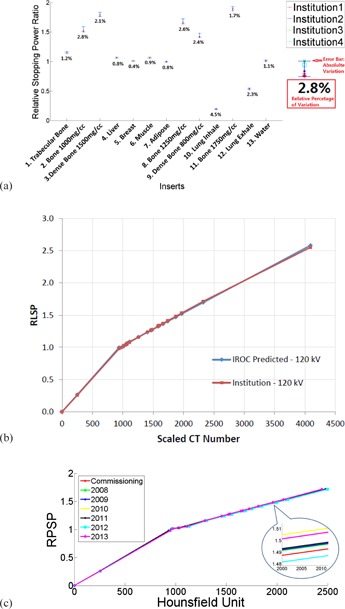
Variations in the stopping power ratios (a) of the CIRS phantom from four proton institutes. The error bars indicated the absolute range of the variations and the digits under the error bars were the percentage variations with respect to the mean values. (b) CT calibration from our institute (red) plotted against the prediction from IROC. (c) CT calibration generated with historic annual QA data from 2008 to 2013.

Four operating proton therapy facilities participated in this study. As demonstrated in Fig. 2(a), reproduced CT calibration curves agreed well in general in the soft‐tissue region with maximum deviation of 1.1% from the average, but deviated more significantly in the bone region with variation up to 2.8%. Range uncertainty from the deviation was determined to be 0.7%±0.2% in our lung cases, and 0.9%±1.2% in brain cases.

Our CT calibration curve is plotted against the one predicted by IROC from their site visit six months after the first patient in Fig. 2(b). Maximum deviation was measured to be only 1.2%.

Calibration curves generated with annual QA data demonstrated tight variations from 2008 to 2013, as shown in Fig. 2(c). The absolute variation (maximum ‐ minimum) in relative stopping power ratios was measured to be 0.019 in the span of six years in a hard bone insert with physical density of 1.25 g / cm^3^, or 1.27% with respect to the mean value.

### B. Beam data

**Figure 3:**
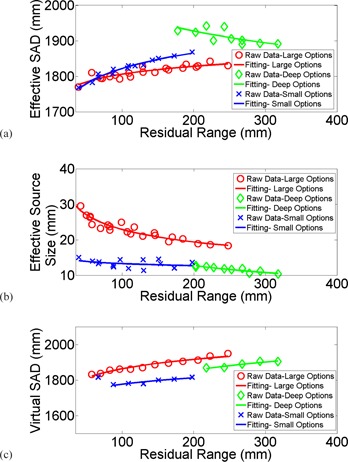
Measurements were plotted against fitting for (a) effective SAD; (b) effective source size; (c) virtual SAD.

Figure 3 shows the effective SADs, effective source sizes, and virtual SADs for all options, plotted against fitting curves. Options sharing the same secondary scatter were grouped into three distinct groups. The effective SAD ranged between 177 cm and 183 cm for large options, 189.1 cm and 192.8 cm for deep options, and 176.8 cm and 186.8 cm for small options. The effective source size ranged between 1.8 cm and 2.9 cm for large options, 1.0 cm and 1.3 cm for deep options, and 1.3 cm and 1.5 cm for small options. The virtual SAD ranged between 183.2 cm and 195.1 cm for large options, 187.0 cm and 190.5 cm for deep options, and 177.5 cm and 181.7 cm for small options.

**Figure 4:**
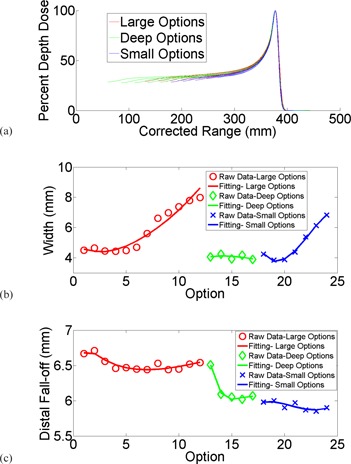
Depth‐dose curves (a) of all 24 options; (b) widths of the pristine Bragg peaks plotted against fitting; (c) distal penumbra plotted against fitting.

The measured pristine Bragg peaks of all 24 options are plotted in Fig. 4. The curves were corrected for the beam divergence and independent of any specific beamline parameters. All overlapped for comparison. Widths of the pristine Bragg peaks varied between 3.9 mm and 8.0 mm, and distal penumbra varied between 5.9 mm and 6.7 mm as plotted for all options in Fig. 4(b) and 4(c). Fitting curves to demonstrate the trend with options in large, deep, and small bands were plotted as well.

### C. Dosimetric commissioning

**Figure 5:**
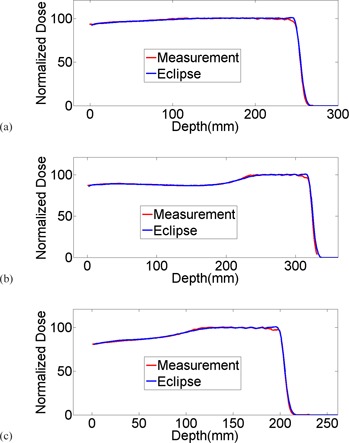
Examples of SOBP measurement plotted against TPS modeling for options 1, 13, and 18; each are options with largest range in large, deep, and small groups.

Examples of SOBP measurements are plotted against TPS modeling for option 1, 13, and 18 in Fig. 5, each of which possesses the largest range for the large, deep, and small bands. Prediction from modeling agreed very well with average 1D gamma rate 95.7%, ranging between 91.8% and 100%. Slight discrepancies on the order of 2% were observed near the distal falloff due to the soft distal shoulder systematically presented for all options. Discrepancies in SOBP between measurements and TPS modeling are summarized in Table 1.

**Figure 6:**
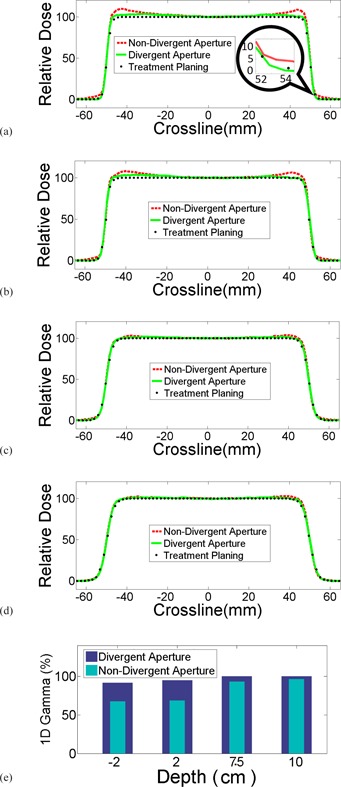
Comparison of crossline profiles of divergent aperture, nondivergent aperture, and treatment planning at various depths. Noticeable differences were observed along the field edges, both inside and outside of the fields. The differences vanished with increased depth in water.

Examples of profile measurements are shown in Fig. 6. All measurements were taken at the nominal SAD. Red lines were crossline profiles for a proton beam with range 15 cm and modulation width 10 cm under a nondivergent block. Collimate scatter was observed on both shoulders at shallow depths. The magnitude of the collimator scatter tapered off with depth. After changing to divergent apertures with inner surface in perfect alignment with the beam divergence, the measured profiles (green lines) agreed very well with TPS modeling (black dots). The passing rate of Gamma analysis increased as well, as demonstrated in Fig. 6(d). Divergent apertures are now used routinely in our clinic.

**Figure 7:**
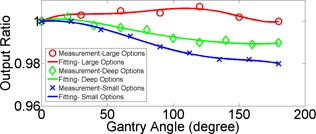
Angular dependency of output in large (red), deep (green), and small (blue) options.

Measurements on the dependence of output on gantry angles are plotted in Fig. 7. The maximum variation was measured 0.7% in large options, 1.1% in deep options, and 2.0% in small options. As the maximum variation was less than 1% in large options, MU was corrected for gantry angle only for fields involving small and deep options in our clinic.

**Table 1:** Summary of SOBP comparisons of measurements vs. modeling

Option	*Range (mm)*	*Modulation (mm)*	*Range Diff. (mm)*	*Gamma (3%/1mm)*	Option	*Range (mm)*	*Modulation (mm)*	*Range Diff. (mm)*	*Gamma (3%/1mm)*
1	250	200	0.30	96.7%	13	320	100	0.28	96.6%
2	225	200	0.13	96.5%	14	295	100	0.28	96.9%
3	208	200	1.46	94.3%	15	270	100	0.07	96.7%
4	187	187	0.60	96.3%	16	245	100	0.04	99.8%
5	167	167	0.61	96.5%	17	220	100	0.26	91.8%
6	148	148	0.60	93.6%	18	200	200	0.68	96.7%
7	131	131	0.68	92.6%	19	177	177	1.24	99.8%
8	114	114	0.00	93.9%	20	153	153	0.46	96.3%
9	99	99	0.07	99.1%	21	132	132	1.27	92.9%
10	85	85	1.07	93.5%	22	110	110	0.89	93.6%
11	72	72	0.38	94.3%	23	90	90	0.29	95.9%
12	60	60	0.74	100%	24	69	69	0.08	92.1%

**Figure 8:**
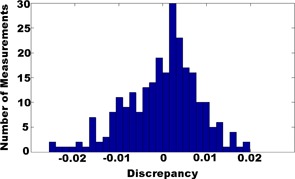
Distribution of discrepancies between our MU prediction model and measurements.

Discrepancies of the output predicted by our MU prediction model and measured with the FC65 chamber in a water tank for the first 400 fields are plotted in Fig. 8. The maximum discrepancy was measured −2.60%. The mean discrepancy was 0.53%.

**Figure 9:**
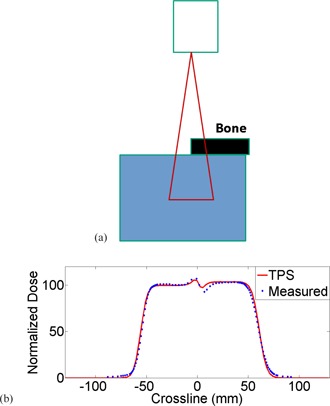
The heterogeneous phantom (a) used for validation of dose distribution. The thickness of the bone slab was 2 cm and the stopping power ratio was 1.63. The measured crossline profile (b) was plotted against prediction from TPS. The maximum discrepancy was measured 4.7%.

Figure 9(a) shows the phantom used to evaluate dose distribution under heterogeneous conditions. The measurement was taken at the nominal SAD with a depth of 27 cm in the water tank. Measured crossline profile under the bone‐air interface is plotted against prediction from TPS in Fig. 9(b). Maximum discrepancy was measured to be 4.7% right under the bone‐air interface. 1D gamma rate (3%/1 mm) for this measurement was 94.6%.

Once we hit the six‐month milestone for treating patients and had treated at least three different disease sites, the IROC Houston group performed a two‐day review of our system including independent measurement of absolute dose, profiles and percent depth dose, and CT calibration curves along with imaging verification accuracy. The output measured by TLDs was within 1% of our institution's designated output. The beam parameters including range, modulation width, flatness, and symmetry were all within the tolerance. The site visit revealed no issues.

In addition, four IROC phantoms have been irradiated and deemed to pass for spine, brain, prostate, and lung. The phantom end‐to‐end testing utilized the same personnel (physicists, dosimetrists, and therapists) as for any patient to conduct simulation, contouring, treatment planning, plan review, MU measurement, QA, and delivery. The results and criteria are summarized in Table 2; all deemed pass as assessed by TLD and film measurement.

**Table 2:** Summary of IROC phantom irradiation results for spine, prostate, head, and lung

*Phantom*	*Location*	*IROC‐H vs. Inst*.	*Criteria*
Spine	TLD Superior	0.96	0.93‐1.07
	TLD Inferior	0.96	0.93‐1.07
Prostate	Location	IROC‐H vs. Inst.	Criteria
	Center Prostate (Left)	0.95	0.89‐1.03
	Center Prostate (Right)	0.94	0.89‐1.03
	Film Plane	Gamma Index	Criteria
	Coronal	99	≥85%
	Sagittal	100	≥85%
Head	Location	IROC‐H vs. Inst.	Criteria
	Target TLD (Right)	0.95	0.95‐1.05
	Target TLD (Left)	0.97	0.95‐1.05
	Film Plane	Gamma Index	Criteria
	Coronal	99%	> 85%
	Sagittal	96%	≥85%
Lung	Location	IROC‐H vs. Inst.	Criteria
	Target Superior	0.95	0.92‐1.02
	Target Inferior	0.96	0.92‐1.02
	Film Plane	Gamma Index	Criteria
	Axial	92%	≥80%
	Coronal	88%	≥80%
	Sagittal	94%	≥0%
	Average over 3 planes	91%	≥85%

### D. Imaging guidance and 6D robotic couch

**Figure 10:**
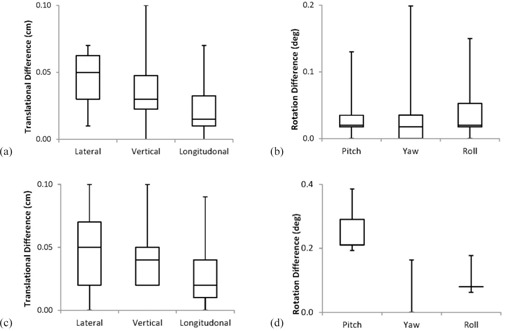
Differences between Verity applied 6D couch corrections and physically measured (a) translations and (b) rotations; results show accuracy is less than 1 mm and 0.2°. Differences between Verity suggested shifts/rotations and known CT (c) translations and (d) rotations; results show accuracy is less than 1 mm and 0.2°.

**Figure 11:**
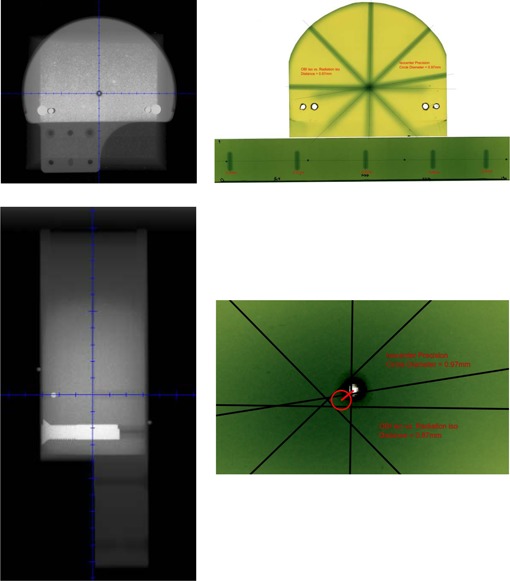
Dedicated star shot phantom kV radiographs (left) and resulting radiochromic couch star shot used for calculating radiation isocenter precision (<1 mm) and distance between imaging and radiation isocenters (<1 mm).

The Verity suggested couch corrections and known CT shifts/rotations agreed within ±1 mm (average: Δlat=0.5 mm; Δvert=0.4 mm; Δlong=0.3 mm) and $\pm 0.4^{\circ }$ (average: $\Delta \text{pitch}=0.24^{\circ }$; $\Delta \text{roll}=0.01^{\circ }$; $\Delta \text{yaw}=0.10^{\circ }$), as demonstrated in Fig. 10. Physical couch measurements and Verity applied corrections agreed within ±1 mm (average: Δlat=0.5 mm; Δvert=0.4 mm; Δlong=0.2 mm) and $\pm 0.2^{\circ }$ (average: $\Delta \text{pitch}=0.03^{\circ }$; $\Delta \text{roll}=0.04^{\circ }$; $\Delta \text{yaw}=0.04^{\circ }$). The directionality of all translations and rotations were qualitatively verified. Figure 11 shows the KV images taken for the couch star shot. The image vs. radiation isocenter coincidence was <1 mm and radiation isocenter precision was <1 mm over the 180° of couch motion, as indicated by film analysis. These tests are conducted monthly.

### E. Initial clinical experience

The majority of treatments (54/100) in our proton center involved brain and central nervous system (CNS) out of the first 100 patients who had completed their treatments by December 2014. Among the 54 patients, 22 were pediatric patients, of whom 8 underwent craniospinal irradiation (CSI) under anesthesia. Other sites that had been treated were lung (26), prostate (6), pelvis (4), head and neck (4), esophagus (4), and GI/liver (2). We currently treat 20‐24 patients per day and up‐time for most months has been better than 95%.

## IV. DISCUSSION

The commissioning process and initial clinical experience were summarized in this study. With the lack of an energy selection system, the Mevion S250 system utilizes a coarse absorber in 5 mm step and a fine absorber in 1 mm step to degrade protons to designed energy all the way down from 250 MeV. This design simplified the system and reduced the cost while offered some unique features: (i) all proton fields, regardless of range, modulation width and option, were measured with similar distal penumbra of 6.3 mm±0.3 mm, and (ii) the ratio of peak to entrance dose is higher in Mevion S250 than other systems due to the inelastic secondary particles generated in the beamline.

An outlier was observed in the derived VSAD of option 24 which didn't follow the fitted trend. Option 24 has the least range for all small options. We believe that this outlying data point was caused by the lack of lead for scatter compensation in the range modulation wheel exclusively used by this option. This range modulation wheel is the only one without lead in all 14 wheels.

Profiles were taken for a beam with range 6.9 cm and full modulation width at various depths to verify our modeling of VSAD for option 24. The discrepancy in field size between measurements and TPS prediction was less than 0.5 mm, predominantly from measurement noise. No systematic deviation was observed.

## V. CONCLUSIONS

The Mevion S250 has been fully commissioned and is in clinical operation in the S. Lee Kling Proton Therapy Center. Its characteristics as a compact single‐room unit are well suited for our requirements on space and budget. The KV imaging is tightly integrated with a 6D robotic couch. Some unique features that come with the design of the system, such as the output dependency on gantry angle and lack of energy selection system, have been investigated and incorporated into our MU model. A variety of sites have been treated, including brain and spine tumors, lung, and other tumor sites. We passed the four IROC credentialing phantoms, complementing our phantom based end‐to‐end testing. Clinically, the system operates well and has provided an excellent system for the treatment of diseases with protons.

## ACKNOWLEDGMENTS

The authors would like to thank Dr. Charles Bloch for his early work on the Mevion S250 system. The authors would also like to thank Dr. Stefan Both, Dr. Christopher Ainsley, Dr. Yuanshui Zheng, Mr. Richard Wu, and Dr. Ronald X. Zhu for their help with the CT calibration measurements.

## COPYRIGHT

This work is licensed under a Creative Commons Attribution 4.0 International License.
